# The brown adipocyte differentiation pathway in birds: An evolutionary road not taken

**DOI:** 10.1186/1741-7007-6-17

**Published:** 2008-04-21

**Authors:** Nadejda V Mezentseva, Jaliya S Kumaratilake, Stuart A Newman

**Affiliations:** 1 Department of Cell Biology and Anatomy, New York Medical College, Valhalla, NY 10595, USA; 2 Discipline of Anatomical Sciences, University of Adelaide, Adelaide, SA 5005, Australia

## Abstract

**Background:**

Thermogenic brown adipose tissue has never been described in birds or other non-mammalian vertebrates. Brown adipocytes in mammals are distinguished from the more common white fat adipocytes by having numerous small lipid droplets rather than a single large one, elevated numbers of mitochondria, and mitochondrial expression of the nuclear gene UCP1, the uncoupler of oxidative phosphorylation responsible for non-shivering thermogenesis.

**Results:**

We have identified *in vitro *inductive conditions in which mesenchymal cells isolated from the embryonic chicken limb bud differentiate into avian brown adipocyte-like cells (ABALCs) with the morphological and many of the biochemical properties of terminally differentiated brown adipocytes. Avian, and as we show here, lizard species lack the gene for UCP1, although it is present in amphibian and fish species. While ABALCs are therefore not functional brown adipocytes, they are generated by a developmental pathway virtually identical to brown fat differentiation in mammals: both the common adipogenic transcription factor peroxisome proliferator-activated receptor-γ (PPARγ), and a coactivator of that factor specific to brown fat differentiation in mammals, PGC1α, are elevated in expression, as are mitochondrial volume and DNA. Furthermore, ABALCs induction resulted in strong transcription from a transfected mouse UCP1 promoter.

**Conclusion:**

These findings strongly suggest that the brown fat differentiation pathway evolved in a common ancestor of birds and mammals and its thermogenicity was lost in the avian lineage, with the degradation of UCP1, after it separated from the mammalian lineage. Since this event occurred no later than the saurian ancestor of birds and lizards, an implication of this is that dinosaurs had neither UCP1 nor canonically thermogenic brown fat.

## Background

Most mammalian species have two types of adipose tissue, white and brown fat, both of which contain adipocytes that store lipids for the production of energy. White adipocytes (WAs) generate metabolically useful energy, while brown adipocytes (BAs) dissipate energy in the form of heat and are responsible for non-shivering thermogenesis [1]. White and brown fat differ in morphology as well as function. Mature WAs contain a single lipid droplet ('unilocular'), few mitochondria, and a nucleus which is displaced to the cytoplasmic periphery. BAs employ numerous mitochondria in heat production. Their lipid is packaged in multiple droplets ('multilocular') and their nuclei are centrally located. The inner mitochondrial membrane of BAs contains uncoupling protein 1 (UCP1). While an alternative non-shivering thermogenesis pathway has been proposed recently for mammalian brown adipocytes [2], in canonical non-shivering thermogenesis heat production depends entirely on UCP1, which facilitates proton leakage and short-circuits oxidative phosphorylation [3].

Although birds can maintain their body temperature by non-shivering thermogenesis and form multilocular adipocytes in response to cold acclimation [4,5] they lack functional BAT [4,6]. Expression of UCP1 is the hallmark of BAs, being absent in WAs, and is the only member of the UCP multigene family to have unambiguous uncoupling activity under physiological conditions [3]. No homologs of this gene have been identified in any bird species [7] (although birds have the paralogous avUCP [8]). While UCP1 is present in both fish [9] and amphibians [10], its function in cold-blooded animals is unknown.

Most studies on adipogenesis have focused on the development of white adipose tissue. WA differentiation has three distinct steps. First, mesenchymal stem cells transform into committed preadipocytes, which have a fibroblastic morphology. Second, white preadipocytes divide until cell-cell contact inhibits their proliferation [11,12]. Third, in the presence of the appropriate differentiation factors (in *in vitro *studies), these preadipocytes enter the cell cycle again, divide at least two more times and finally differentiate into WAs [11,12].

Terminal differentiation of WAs is preceded by the induction of CCAAT/enhancer binding proteins beta (C/EBPβ) and delta (C/EBPδ), which in turn mediate the transcriptional activation of peroxisome proliferator-activated receptor γ (PPARγ) and C/EBPα. PPARγ is a key factor of WA differentiation [11,12] and its forced expression in non-adipogenic fibroblasts is sufficient to induce adipogenesis [13]. Thus, PPARγ plays a crucial role in the expression of many, and perhaps most, fat-cell-specific genes [12], including those specifying adipocyte-specific fatty acid-binding protein (FABP4) [14] and other proteins involved in lipid homeostasis [12].

While less is known about BA differentiation, several steps occur in common with white adipogenesis. During the initial steps, the mesenchymal progenitor cells become committed as fibroblast-like preadipocytes. Although these cannot be distinguished morphologically from white preadipocytes [12] their gene expression signature is already different from the latter at this stage [15]. The key WA differentiation transcriptional factor, PPARγ, is also involved in BA differentiation [16-18]. However, while PPARγ is necessary and sufficient to induce the WA differentiation program, it is necessary but not sufficient for BA differentiation. In addition, a PPARγ interacting protein, peroxisome proliferator-activated receptor γ coactivator 1α (PGC-1α), which is expressed preferentially in BAs relative to WAs, is required to generate the former cell type [19]. In muscle cells, PGC-1α mediates the increase in mitochondria by stimulating gene expression of nuclear respiratory factors (NRF) 1 and 2, and consequently mitochondrial transcriptional factor A (mitTFA) [20].

Here we report that a stem cell-like mesenchymal population derived from embryonic avian limb buds can be induced to differentiate into BA-like cells *in vitro*. We show that whereas UCP1 is absent from the chicken genome [10,21], foreclosing the development of authentic BAs, the conditions that induce avian BA-like cells (ABALCs) elicit expression of a virtually complete mammalian-like BA differentiation pathway, to the extent that transfected mouse UCP1 promoter is strongly activated. The unusual circumstance of a gene being present in a conserved chromosomal context in fish, amphibians and mammals, but not birds, prompted us to look for it in the same context in the recently mapped *Anolis *lizard genome, where it was also absent. This suggests that the gene, and the capacity to produce brown fat, were lost no later than in the saurian common ancestor of birds and lizards, a lineage that also included all theropod dinosaurs.

## Results and Discussion

### Brown adipocyte-like morphology in cultured limb bud mesenchyme

**Figure 1:**
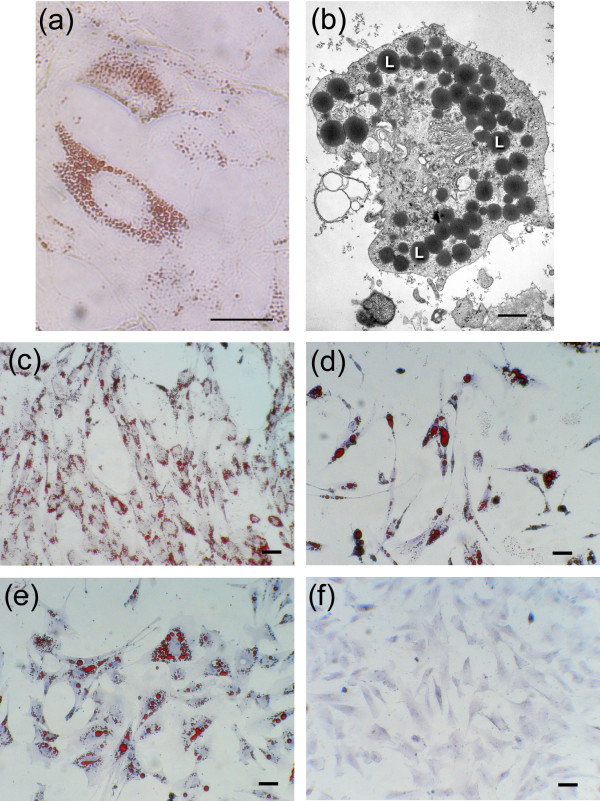
**Appearance of cells with mammalian brown fat-like phenotype in embryonic chicken mesenchyme *in vitro***. **(a) **Oil-red and hematoxylin staining of light micrographs of limb bud mesenchyme (LBM) cells grown in adipocyte differentiation medium (ADM) for 22 days with 10% horse serum. **(b) **Transmission electron micrograph of a single cell from 22-day culture as in (a); L, lipid droplet. **(c)-(f) **Oil-red and hematoxylin staining of light micrographs of 8-day cell cultures.**(c) **Adipocytes from LBM cells grown in ADM with 10% horse serum. **(d) **Adipocytes from vascular stromal cells (VSCs) grown in ADM with 10% horse serum. **(e) **Adipocytes from LBM grown in ADM with 10% chicken serum. **(f) **LBM cells grown in ADM with 10% FBS. Scale bars: (a) 10 μm, (b) 1 μm; (c)-(f) 50 μm

The distal tip mesoderm of the embryonic chicken limb bud at 5 days of incubation contains no cells of the myogenic lineage [22,23], but this limb bud mesenchyme (LBM) can be induced to differentiate into cartilage [24,25] or fibroblasts [26] by growth under different density and culture conditions. When grown at low density in adipocyte differentiation medium (ADM) containing horse serum (HS), LBM gave rise to a cell population which contained numerous small Oil-red positive lipid droplets (Figure 1a, c), which did not fuse even after 22 days in cell culture (Figure 1a, b). In contrast, vascular stromal cells (VSCs), a characterized population of chicken white fat preadipocytes [27], grown under the same conditions, gave rise to cells containing a few large droplets of Oil red-staining lipid, which fused, forming even larger droplets (Figure 1d).

Both cell origin and treatment regimen were critical for producing what we term ABALCs. When LBM cells were grown in ADM with chicken serum (CS), for example, they differentiated into cells with the WA phenotype (Figure 1e), and when they were grown in ADM with fetal bovine serum (FBS) they showed no adipocyte-like morphological features (Figure 1f). Significantly, cells grown in FBS for 8 days nonetheless constituted a bipotential preadipocyte population, since when they were transferred into a medium containing CS or HS they uniformly differentiated into WAs or ABALCs, respectively (data not shown). We therefore refer to them as limb bud-derived preadipocytes (LBPAs).

**Figure 2:**
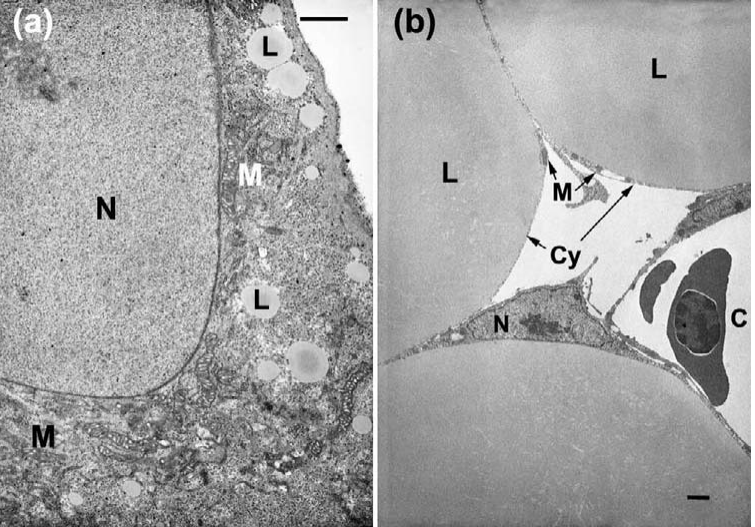
**Transmission electronmicrographs of adipocytes**. **(a) **Adipocyte from limb bud mesenchyme (LBM) grown in adipocyte differentiation medium (ADM) with 10% horse serum for 22 days. **(b) **Adipocyte from the abdominal fat of a 20-day chicken embryo. Cy, cytoplasm; C, capillary; N, nucleus; M, mitochondrion; L, lipid droplet. Scale bars: 1 μm.

Transmission EM was used to compare the morphology of ABALCs with WAs obtained from the abdominal fat pads of 20-day chicken embryos. ABALCs were irregularly shaped and had many cell processes (Figure 2a). Their nuclei were relatively large and not compressed to the periphery by the lipid droplets as in abdominal fat cells, in which the lipid droplets occupied most of the cell space (Figure 2b). Cross-sectional area measurements [28,29] indicated that ABALCs were smaller than abdominal fat pad WAs (Table 1). The mean volume of a mitochondrion was similar in both ABALCs and WAs, but mean mitochondrial volume density (Vv) was five-fold higher in ABALCs than in WAs (Tables 2 and 3). The mean volume of ABALC lipid droplets was significantly smaller than those of abdominal fat cells, while the number density of these lipid droplets was much higher than that in WAs (Table 3). Measurements of the ABALC lipid droplets revealed that about 98% had cross-sectional areas of less than 1.5 μm^2^, while the areas of the rest ranged from 1.5 to 7.0 μm^2 ^(Additional file 1). In contrast, the cross-sectional areas of 50% of the lipid droplets in WAs were larger than 7.0 μm^2^, and ranged from 7.0 to 1,262.2 μm^2 ^(Table 1 and Additional file 1). These dramatic morphological differences between ABALCs and chicken WAs, as well as qualitative similarities between ABALCs and mammalian BAs, motivated us to take a closer look at these presumptive avian 'brown fat' cells.

**Table 1:** Mean cross-sectional areas of adipocytes and lipid droplets in ABALCs and white fat adipocytes

Cell type	Cross sectional area (μm^2^)		
	
		Lipid droplets	
		
	Whole cell	Mean	Range
ABALCs	191.8 ± 8.2*	0.40 ± 0.01*	0.031–6.626
Abdominal fat	716.7 ± 35.4*	51.7 ± 9.7*	0.283–1,262.215

*Significantly different at α = 0.05. One-way ANOVA; Kruskal-Wallis on ranks.

**Table 2:** Mean volume density of cytoplasm, nuclei, mitochondria and lipid droplets in ABALCs and white fat adipocytes

	Volume density (μm^3^/100 μm^3 ^of cell)			
	
Cell type	Cytoplasm	Nuclei	Mitochondria	Lipid droplets
ABALCs	73.17 ± 4.1*	7.88 ± 2.5	4.18 ± 0.58*	14.8 ± 3.9*
Abdominal fat	5.91 ± 1.0*	2.44 ± 1.1	0.71 ± 0.14*	90.9 ± 1.6*

*Significantly different at α = 0.05. One-way ANOVA; Kruskal-Wallis on ranks.

**Table 3:** Mean volume and number density of mitochondria and lipid droplets in ABALCs and white fat adipocytes

	Volume of a single organelle (μm^3^)		Number density (per μm^2 ^cell)	
	
Cell type	Mitochondrion	Lipid droplet	Mitochondria	Lipid droplet
ABALCs	0.72 ± 0.14	1.88 ± 0.4*	0.076 ± 0.012*	0.11 ± 0.02*
Abdominal fat	0.52 ± 0.16	788 ± 289*	0.017 ± 0.003*	0.002 ± 0.0003*

*Significantly different at α = 0.05. One-way ANOVA; Kruskal-Wallis on ranks.

### ABALCs utilize common pathways of adipocyte differentiation

**Figure 3:**
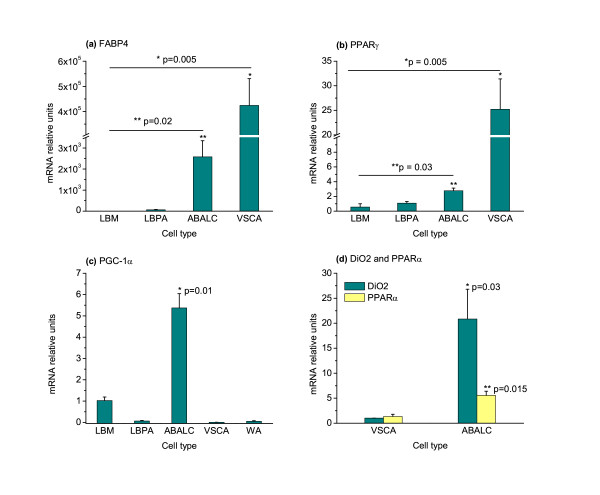
**Avian brown adipocyte-like cells express common adipocyte markers and specific brown adipocyte markers**. Quantitative reverse-transcription polymerase chain reaction (qRT-PCR) analysis of relative expression of genes common to differentiated phenotype of white and brown adipocytes and specific to brown fat differentiation in limb bud mesenchyme (LBM), limb bud preadipocytes (LBPAs), avian brown adipocyte-like cells (ABALCs) in 8-day cell culture, vascular stromal cells adipocytes (VSCAs) and white adipocytes (WAs) from abdominal fat of 20-day chicken embryos. **(a) **Fatty acid binding protein, FABP4. **(b) **Peroxisome proliferator activated receptor γ, PPARγ. **(c) **Peroxisome proliferator-activated receptor γ coactivator 1α, PGC-1α. **(d) **Type II iodothyronine deiodinase, DiO2 and peroxisome proliferator activated receptor α, PPARα. Each treatment was repeated three times. Measurements were in triplicate and normalized to β-actin mRNA levels. Relative mRNA levels were analyzed by one-way analysis of variance and means were compared using the Tukey test. Results are shown as means ± standard error of the mean.

As assayed by comparative quantitative reverse-transcription polymerase chain reaction (qRT-PCR), both PPARγ and FABP4 mRNAs were expressed in ABALCs. The levels of expression of each gene were significantly higher in ABALCs than in LBPAs and in progenitor LBM, although they were highest in WAs differentiated from VSCs in culture (Figure 3a, b). Oil-red positive lipid staining, together with increased levels of PPARγ and FABP4, indicate that ABALCs are indeed adipocytes.

### ABALC induction involves pathways specific to BA differentiation

We found that expression of PGC-1α mRNA was 5 to 10 times higher in ABALCs than in LBM, LBPAs, WAs obtained from VSCs (VSCAs) *in vitro*, or late embryonic white fat cells (Figure 3c). Similarly, the expression of mRNA for PPARα and DiO_2 _(Type II iodothyronine deiodinase), additional proteins previously found to be upregulated in BAs relative to their preadipocytes or WAs [30,31], was increased in ABALCs in comparison to VSC adipocytes (Figure 3d). Thus, ABALCs not only resemble mammalian BAs morphologically but also with respect to their molecular signature.

**Figure 4:**
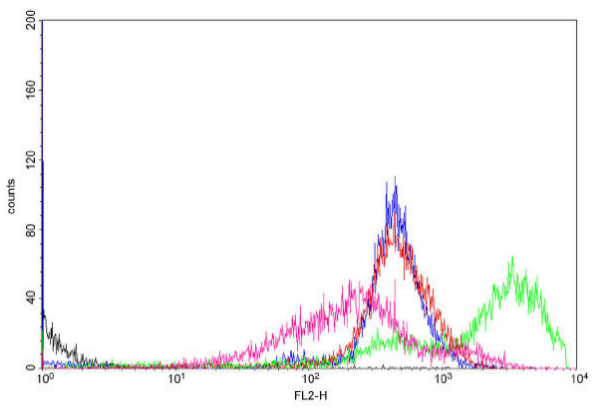
**ABALC cellular phenotype is associated with increased mitochondrial volume**. FACScan of cells stained with JC-1. FL2-H is red fluorescence. Black line, unstained mesenchymal cells; blue line, stained mesenchymal cells; red line, limb bud preadipocytes (LBPAs); pink line, limb bud mesenchyme (LBM) adipocytes grown in chicken serum; green line, avian brown adipocyte-like cells (ABALCs).

**Figure 5:**
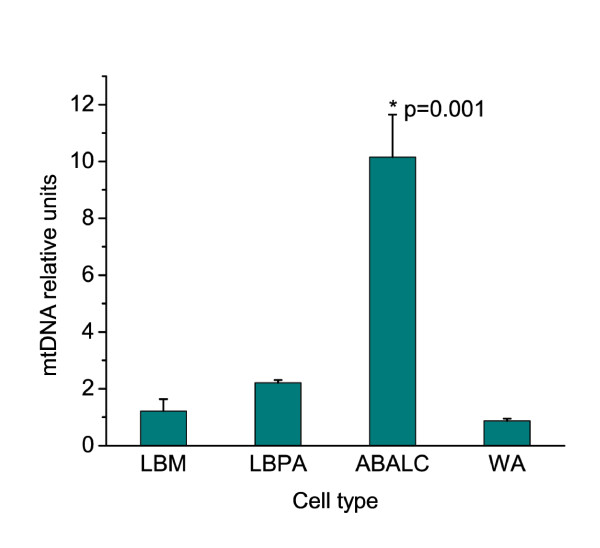
**Quantitative polymerase chain reaction analysis of relative mtDNA levels**. Each measurement was normalized to β-actin DNA levels. Relative mtDNA levels were analyzed using one-way analysis of variance comparing means by the Tukey test. Results are shown as means ± standard error (*N *= 3)

The ability to quantify PGC-1α mRNA expression provided an opportunity to assay the relationship between the expression of this gene and mitochondrial biogenesis in the various cell types of the adipogenic lineage. This required more straightforward means for measuring mitochondrial parameters than the morphometric analysis of electron micrographs described above (see Table 2). First we stained LBM, LBPAs, limb bud-derived WAs and ABALCs with JC-1, a dye that accumulates specifically in mitochondria [32]. Flow cytometry demonstrated that ABALCs had the highest red fluorescence per cell among all of these cell types (Figure 4). Although red fluorescence of JC-1 can be indicative of high mitochondrial membrane potential [32], the fact that the red-to-green fluorescence ratio of the ABALC mitochondria was no greater than the ratios of both LBM and LBPAs (data not shown) indicated that the observed elevated red fluorescence was indeed due to increased mitochondrial volume. Next we used quantitative PCR (qPCR) to measure mtDNA and found that ABALCs had about 10 times as many copies of this genome as LBM, LBPAs, or WAs (Figure 5). Thus, ABALCs, which have increased levels of PGC-1α mRNA relative to their progenitor cells, also have increased mitochondrial volume and elevated levels of mtDNA compared with progenitor cells, the bipotential preadipocytes derived from the latter, or the differentiated cells of white fat.

### The chicken and lizard genomes lack the UCP1 gene

**Figure 6:**
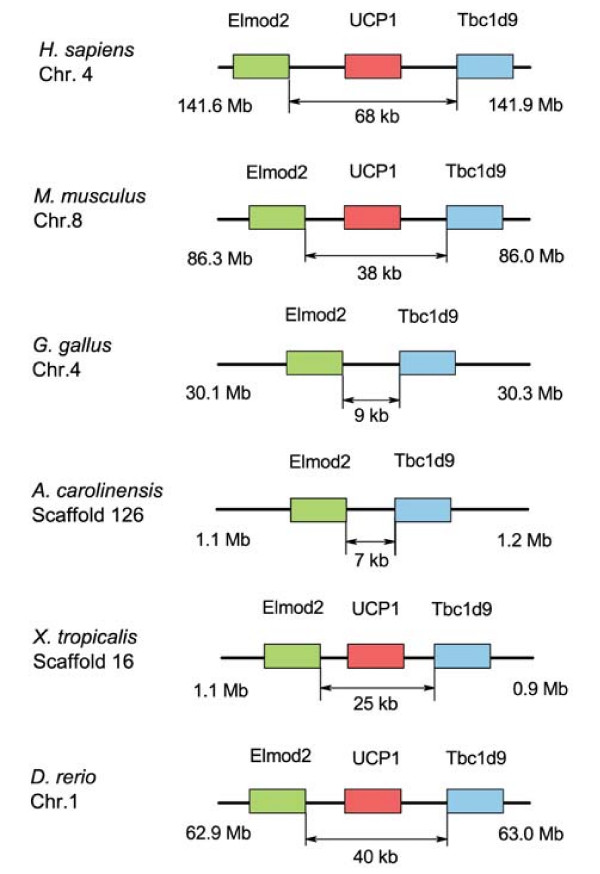
**Genomic comparison of human, mouse, chicken, lizard, amphibian and fish genomes reveals absence of UCP1 gene in chicken and lizard**. Genomic sequences and contexts were analyzed using the Ensembl [42] and University of California Santa Cruz [43] genome browsers.

The hallmark of mammalian BAs is the uncoupler of oxidative phosphorylation UCP1, which gives brown fat the unique ability to produce heat. Neither UCP1 nor a homolog of this gene have been identified in any bird species. Interestingly, UCP1 has been found in the genomes of several fish species [9] and amphibians [10], where it is flanked by the Elmod2 and Tbc1d9 genes, as it is in mammals [9]. We compared the genomic regions containing the UCP1 gene in *Homo sapiens*, the mouse *Mus musculus*, the frog *Xenopus tropicalis*, the lizard *Anolis carolinensis*, and the zebrafish *Danio rerio*, with the corresponding sequences in the chicken *Gallus gallus *(Figure 6). The regions between the Elmod2 and Tbc1d9 genes in the chicken [10,21] and lizard genomes do not contain any open reading frames specifying peptides homologous to UCP1, nor is there significant similarity at the DNA level. Correspondingly, the length of this region is markedly smaller in chicken and lizard compared with the species which have a UCP1 gene (Figure 6 and Table 4). Blast search against the chicken nucleotide database using mouse or *D. rerio *UCP1 mRNA sequences revealed significant similarity only with mRNA sequences of the UCP1 paralog avUCP.

**Table 4:** Comparison of genomic regions flanked by Elmod2 and Tbc1d9 in selected organisms

Organism	Interval between Elmod2-Tbc1d9 (bases)	Number of ORF > 100 aa	UCP1 gene length	Number of exons	Longest exon (bases)	Shortest exon (bases)	Chromosome or scaffold*
Zebrafish	40,123	15	14,435	7	1,592	77	1
Xenopus	25,058	6	3,601	6	208	102	16
Lizard	6,978	1	-	-	-	-	126*
Chicken	8,888	3	-	-	-	-	4
Platypus	64,497	15	14,793	6	199	102	33
Armadillo	22,088	12	5,037	6	201	102	5,996*
Mouse	37,694	15	8,101	6	595	102	8
Human	68,425	15	8,958	6	250	102	4

### Activation of an exogenous UCP1 promoter during ABALC differentiation

**Figure 7:**
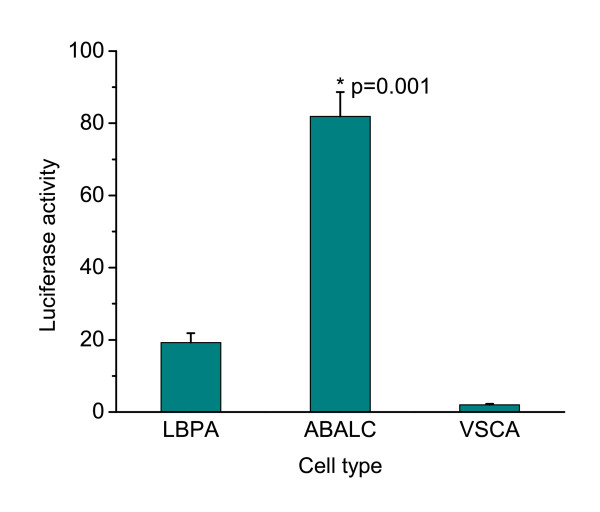
**Mouse uncoupling protein 1 promoter is activated in avian brown adipocyte-like cells but not in vascular stromal cell adipocytes**. Firefly luciferase activity was normalized with Renilla luciferase. Normalized Firefly luciferase activities were compared using one-way analysis of variance using the Tukey test. Results are shown as means ± standard error (*N *= 8).

Despite the lack of the UCP1 gene in the chicken, our findings suggested that cells derived from the limb bud of this species express the necessary regulatory components for the differentiation of BAs. We found that expression of the avian uncoupling protein avUCP was not upregulated, and indeed substantially declined, under ABALC-inducing conditions (data not shown). We reasoned, however, that if ABALCs were similar to mammalian BAs except for the absence of UCP1, differentiating ABALCs should be able to activate an exogenously provided mammalian UCP1 promoter. To test this hypothesis we transfected ABALCs, VSC adipocytes and preadipocytes with a mouse UCP1 promoter-Luciferase construct [33] and assayed relative transcriptional activity in these cell types. We found that the mouse UCP1 promoter was strongly activated in ABALCs and slightly in LBPAs, but not at all in adipocytes obtained from VSCs (Figure 7). The inability of chicken VSC adipocytes to activate the mouse UCP1 promoter is in agreement with the finding that during mammalian WA differentiation *in vivo *UCP1 promoter is never activated [34]. The ability of ABALCs to activate the UCP1 promoter, however, indicates that rather than being an aberrant form of WAs, they contain transcriptional capabilities that correspond to their BA-like morphological phenotype.

### Conclusion and evolutionary implications

We have shown that adipocytes with the brown fat phenotype, termed ABALCs, can be induced in embryonic chicken mesenchymal cells *in vitro*. ABALCs not only share morphology with mammalian BAs but also express the brown fat regulatory protein PGC-1α, have increased mitochondrial genome number and produce all necessary factors for mammalian UCP1 promoter activation.

While it has been suggested that BAs and WAs originate from a common population of mesenchymal cells, it is not known how and at what stage the differentiation pathways diverge from one another. The view that BAT and WAT are distinct terminally differentiated tissues is supported by their different functions and morphological features [11] and the fact that precursor cells isolated from brown or white fat differentiate into BAs and WAs, respectively, *in vitro *[35]. Furthermore, BAs and WAs have different 'expression signatures' [15]. It is still not clear, however, if mesenchymal cells of different origin have restricted ability to differentiate into BAs or WAs or, rather, if the location of those mesenchymal cells determines their differentiation fate. Our results confirm earlier findings [34,36] that plasticity of mesenchymal cells is already lost in VSC preadipocytes, since these precursor cells were not able to differentiate into ABALCs in response to the stimulus that elicits this phenotype from LBM.

Most significantly, because LBM can give rise with near uniformity to adipocytes with white and brown fat cellular phenotypes under different culture conditions, our results suggest that LBM may be a source of a common BA and WA stem cell, which up to now has proved elusive [11]. If it can be confirmed that LBM of mammalian species indeed generates both WA and authentic BA, this system would be ideal for studying the relationship between the different adipocyte differentiation pathways, a growing issue in obesity research [11,12].

Results with knockout mice demonstrate that even in mammals the developmental pathway for producing multilocular adipose tissue does not depend on UCP1 [2]. If thermogenic brown fat evolved before the loss of UCP1 during avian evolution, the non-thermogenic brown fat-like tissue that would have remained could have had its own adaptive function. Indeed, all reports of BAT-like tissue in birds and turtles have described it as highly vascularized [5,37,38], suggesting that it can serve as source of fuel which can be rapidly mobilized and transported to other sites (for example, muscles), in the form of fatty acids [39].

**Figure 8:**
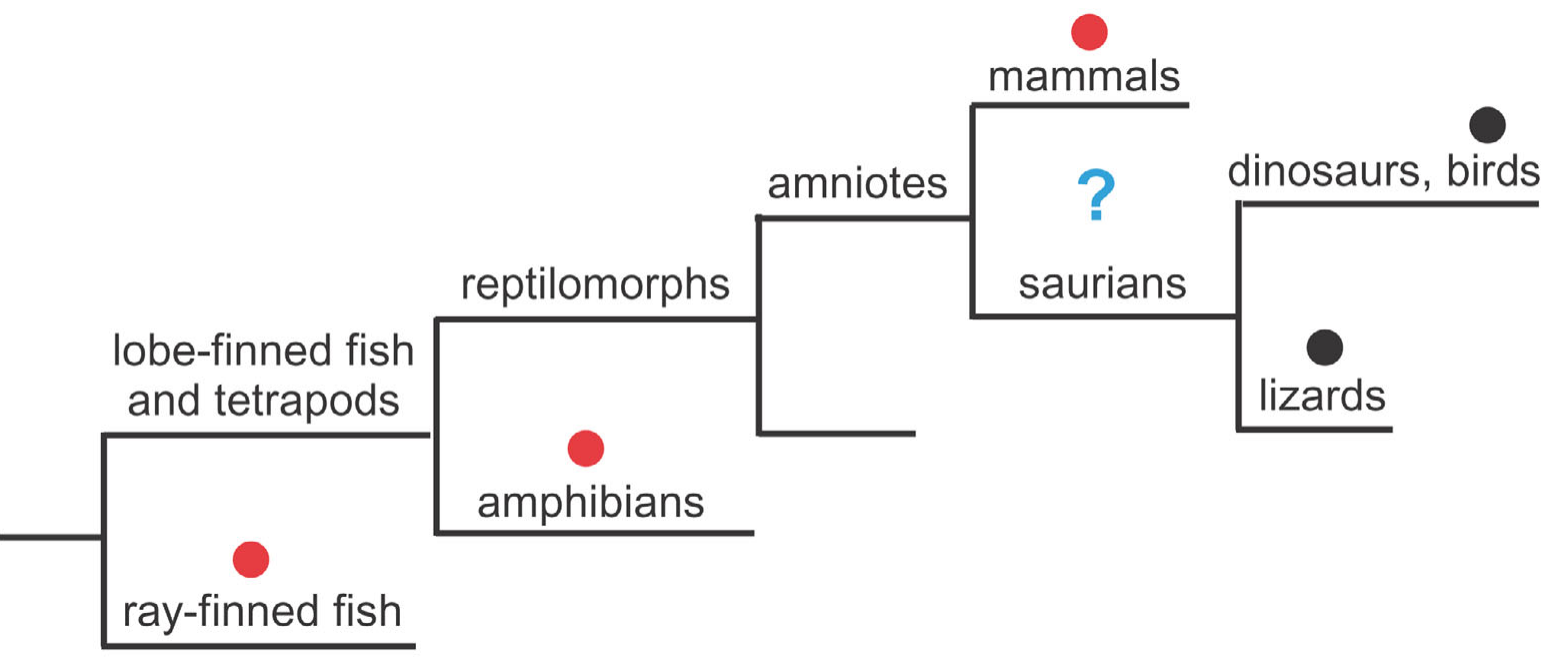
**Partial cladogram of the vertebrates**. Taxa in which UCP1 are known to be present are indicated by a red dot. Those in which UCP1 is known to be absent are indicated by a black dot. The question mark indicates the span within the evolutionary history of the amniotes during which UCP1 and the capacity to produce functional brown fat was lost.

While the UCP1 gene is absent in the chicken, we have demonstrated that the developmental pathway that generates brown fat in mammals is otherwise intact. Attempts to identify UCP1 in avian species other than chickens have been unsuccessful [7]. The most parsimonious explanation for the absence of the UCP1 gene in both the chicken and the *Anolis *lizard is that it was lost in a common saurian ancestor of birds and lizards, or even earlier (Figure 8). Since the saurian line also includes the theropod dinosaurs, it is an all-but inescapable conclusion that dinosaurs had neither UCP1 nor thermogenic brown fat (Figure 8).

Homeotherms, such as mammals and birds, have adaptations that protect developing offspring and young from the ill effects of cold. In mammals, the embryos are mainly incubated internally and brown adipose tissue is particularly abundant at juvenile stages. Oviparous birds and presumably dinosaurs, lacking thermogenic BAT (although, notably, an egg-laying mammal, the platypus, has a UCP1 gene; Table 4), would require other means, which in birds include feathers, brooding incubation, and non-shivering thermogenesis mediated by expression of avUCP in muscles [39,40]. No information is available concerning avUCP in dinosaurs, but it is not likely that these large featherless animals were effective brooders. The lack of thermogenic BAT may thus have been more of a liability to the developing embryos of oviparous dinosaurs than to those of birds. Further investigations of the possible occurrence of the UCP1 gene and the BA pathway in testudine reptiles (that is, turtles and tortoises) should clarify the evolutionary history of brown fat as well as the phylogenetic relationships among modern amniotes.

## Methods

### Cell cultures

Mesenchymal cells were isolated from distal tips of 5-day chicken embryo leg buds as described previously [41]. Cells were dissociated in TrypLE Express solution (Gibco), washed and resuspended for plating 4 × 10^5 ^cells per ml, and seeded in 24-well tissue culture plates in plating medium (M199 containing 10% FBS and 1% PenStrep (Gibco)) at a cell density of 2 × 10^5 ^cells per well. Cells were maintained in plating medium for 24 hours, when adipocyte differentiation was initiated by addition of adipocyte differentiation medium (ADM) (60% DMEM/40% F12, 20 μg/ml insulin, 500 nM dexamethasone, 1% PenStrep, 10% HS or, depending on the experiment, 10% CS or FBS). Preadipocyte cell cultures were grown in plating medium without initiation of differentiation. Vascular-stromal cells were isolated from 30-day postnatal chickens, as described [27]. Cells were dissociated and plated as for mesenchymal cells. Adipocyte differentiation was initiated in these cultures by adding ADM when cells reached about 80% confluence.

### Electron microscopy

Adipocytes grown in ADM with HS for 22 days were scraped off the wells of culture plates, centrifuged at 500 rpm in a microcentrifuge in microfuge tubes to form a soft pellet. The pellet was fixed for 1 hour, gently scooped off the bottom of the tube and further fixed overnight (16 to 18 hours) at 4°C with a chilled (4°C) fixative containing 3% glutaraldehyde, 4% paraformadehyde, 4% polyvinylpyrrolidone (MW 10,000), 4% sucrose and 2 mM CaCl_2_, prepared in 0.1 M cacodylate buffer with a final pH of 7.4. The pellet was post-fixed in 1% Osmium tetroxide for 1 hour, dehydrated through a graded series of ethanol, embedded in Spurr's resin and polymerized at 60°C. In addition, abdominal fat pads from 15-day chicken embryos were fixed, dehydrated and embedded in resin in the same fashion. Thin sections (silver in interface) from the resin blocks were mounted on copper grids, stained with uranyl acetate and lead citrate and examined using JEOL or Hitachi electron microscopes.

### Morphometry

Electron micrographs (24.7 × 18.1 cm^2^) were obtained from random fields at a magnification of × 7,500. The magnification was checked at frequent intervals using a carbon graticule. Volume densities (Vv) of 'cytoplasm' (area of the cell which excludes nucleus, mitochondria, and fat droplets), nucleus, mitochondria, and fat droplets of adipocytes were determined by point counting [29] using a transparent grid with 682 intersections. Number densities (number per μm^2 ^of cell) of mitochondria and fat droplets of the adipocytes were also determined [28,29]. The β values for the calculation of the number density [28,29] of mitochondria and fat droplets were determined by taking measurements from over 275 mitochondria and fat droplets of adipocytes of tissue cultures from each treatment and abdominal fat pads using the NIH Image J program. Cross-sectional areas of fat droplets were determined from electron micrographs, and cross-sectional areas of whole fat cells were determined on Toluidine blue stained semi-thin sections from the same resin blocks, using NIH Image J.

### Oil-red staining

Cell cultures were washed with 1× PBS (phosphate buffered saline, pH 7.4) and fixed for 10 minutes in 4% paraformaldehyde solution in 1× PBS. Cells were then briefly rinsed in 1× PBS and stained with a 0.5% Oil-Red O (Fluka) solution in isopropanol/water (6:4) for 1 hour at room temperature. Cells were washed in 1× PBS and stained with Hematoxylin (Fisher) for 2 minutes and then washed again in 1× PBS. Microscopic pictures were obtained with an Zeiss Axiovert 40C microscope equipped with an Olympus C7070 digital camera.

### Comparative qPCR

Total RNA was isolated from cell cultures or cells with the RNeasy Mini kit (Qiagen) according to the manufacturer's instructions. RNA was reverse transcribed by AMV (Fisher). Briefly, 200 to 1,000 ng of RNA and Oligo dT (0.5 μg primer per 1 μg of RNA) was heated at 70°C for 5 minutes. The tube was chilled on ice for 5 minutes and a master mix containing AMV buffer, dNTPs (1 mM each base), 40 units ribonuclease inhibitor and 30 units AMV RT was added. The reaction was performed at 42°C for 1 hour and stopped by AMV inactivation by heating at 80°C for 3 minutes. Genomic and mitochondrial DNA were isolated using the QIAamp DNA Mini kit (Qiagen). Comparative qPCR was performed on the Mx3005P instrument (Stratagene) using Brilliant SYBR Green QPCR master mix, and following the standard comparative qPCR protocol. All primers (Additional file 2) were designed using the Beacon Designer program (Premier Biosoft, Palo Alto, CA).

### Flow cytometry

Cell cultures were washed with Earle's balanced salt solution (EBSS) and JC-1 dye diluted in 500 μl of DMEM to a final concentration of 2 μM was added to each well. Cells were incubated for 10 minutes in a 5% CO_2 _incubator and washed with EBSS without Ca^2+ ^and Mg^2+ ^and then dissociated in TrypLe Express, counted and resuspended in EBSS with a density of 10^6 ^cells per ml. Freshly isolated mesenchymal cells were resuspended in EBSS with 2 μM JC-1 and placed for 10 minutes in a CO_2 _incubator. The cells were then washed and resuspended in EBSS. The forward and right angle scatter, as well as green and red fluorescence emissions of cultured and mesenchymal cells, were measured in a FACScan flow cytometer (BD Biosciences, San Diego, CA). Data were acquired and analyzed using Cell Quest software (BD Biosciences).

### UCP1 promoter activation assay

A mouse UCP1 promoter pGL/basic construct [33] was a gift from Dr. J Rim and Dr. L Kozak. LBM cells or VSCs were grown in plating medium for 1 day. The transfection complexes containing siPORT-XP-1 (Applied Biosystems), 200 ng of pGL-UCP1 promoter-Firefly Luciferase construct, 20 ng pGL-TK Renilla Luciferase construct (Promega) and OptiMem (Invitrogen) were added on the second day with ADM without antibiotics. Mouse UCP1 promoter activation was estimated on the second day after transfection by the Dual-Luciferase reporter assay (Promega) in a model 20/20 luminometer (Turner Biosystems).

## List of abbreviations

ABALC: avian brown adipocyte-like cell; ADM: adipocyte differentiation medium; BA: brown adipocyte; BAT: brown adipose tissue; C/EBP: CCAAT/enhancer binding protein; CS: chicken serum; DiO_2_: type II iodothyronine deiodinase; FABP4: adipocyte-specific fatty acid-binding protein; FBS: fetal bovine serum; HS: horse serum; LBM: limb bud mesenchyme; LBPA: limb bud preadipocyte; mitTFA: mitochondrial transcriptional factor A; NRF: nuclear respiratory factor; PPAR: peroxisome proliferator-activated receptor; PGC-1α: peroxisome proliferator-activated receptor γ coactivator 1α; qPCR: quantitative polymerase chain reaction; qRT-PCR: quantitative reverse-transcription polymerase chain reaction; UCP1: uncoupling protein 1; VSC: vascular stromal cell; VSCA: vascular stromal cells adipocyte; WA: white adipocyte.

## Authors' contributions

SAN discovered ABALCs in cultures prepared by NVM. SAN and NVM designed, and NVM performed, the experiments. JSK performed the electron microscopy and morphometric analyses. NVM, JSK and SAN wrote the paper.

## Supplementary Material

Additional file 1 Table showing the size distribution of lipid droplets in avian brown adipocyte-like cells and abdominal white fat according to cross-sectional area. Click here for file

Additional file 2 Table showing the set of primers used for quantitative reverse-transcription polymerase chain reaction and quantitative polymerase chain reaction. Click here for file
