# Supplementary material for: The brown adipocyte differentiation pathway in birds: An evolutionary road not taken
Source: BMC Biol. 2008 Apr 21;6:17. doi: 10.1186/1741-7007-6-17 (PMC2375860; doi:10.1186/1741-7007-6-17)
Supplement: Additional file 1 — Table showing the size distribution of lipid droplets in avian brown adipocyte-like cells and abdominal white fat according to cross-sectional area. [file 1741-7007-6-17-S1.doc]

**Additional data file 1**

Size distribution of lipid droplets according to cross sectional area

| Intervals  µm^2^ | Percentage of lipid droplets | |
| --- | --- | --- |
|  | ABALC | Abdominal Fat |
| 0.0 | 0.0 | 0.0 |
| 0.5 | 77.0 | 4.71 |
| 1.0 | 17.47 | 11.23 |
| 1.5 | 3.16 | 5.43 |
| 2.0 | 0.79 | 7.97 |
| 2.5 | 0.63 | 5.43 |
| 3.0 | 0.24 | 3.62 |
| 3.5 | 0.32 | 1.81 |
| 4.0 | 0.16 | 0.73 |
| 4.5 | 0.0 | 1.45 |
| 5.0 | 0.0 | 2.17 |
| 5.5 | 0.16 | 2.54 |
| 6.0 | 0.0 | 2.54 |
| 6.5 | 0.0 | 0.73 |
| 7.0 | 0.08 | 0.73 |
| >7.5 | 0.0 | 49.64 |
