# Supplementary material for: The brown adipocyte differentiation pathway in birds: An evolutionary road not taken
Source: BMC Biol. 2008 Apr 21;6:17. doi: 10.1186/1741-7007-6-17 (PMC2375860; doi:10.1186/1741-7007-6-17)
Supplement: Additional file 2 — Table showing the set of primers used for quantitative reverse-transcription polymerase chain reaction and quantitative polymerase chain reaction. [file 1741-7007-6-17-S2.doc]

# Additional data file 2

Primer sets used for qRT-PCR and qPCR

| Gene of interest | Accession number | Forward primer (5’ to 3’) | Reverse primer (5’ to 3’) |
| --- | --- | --- | --- |
| β-actin | NM205518 | CGGTACCAATTACTGGTGTTAGATG | GCCTTCATTCACATCTATCACTGG |
| PPARγ | AF163811.1 | TGTCGCATCCATAAGAAAAGCAG | GATGTCGCTGGAAATCTCTGC |
| FABP4 | NM204290.1 | GATCCTGTGAAAGACTGCTACCTG | ACCATTGATGCTGATAGTTAAATTAG |
| PGC-1α | NM001006457 | GTACAGCGACCAGTCTGAGG | CAAGTTTGCCTCATTCTCTTCATC |
| PPARα | NM001001464.1 | CGATATCTTCCTCTTCCCGAAAC | GTAAAGGGTGTAAATGTGCATCAG |
| DiO_2_ | NM204114.1 | GAGGCTAATACAGAGTTATGAGAC | AAGACCAGGAGATGAGAATGAC |
| Mit DNA | X52392.1 | GCACAAACGCTTAAAACTCTAAGG | TTGGGTGAATCGTGGATTATCG |
